# Protective Assets Reinforced With Integrated Care and Technology (PARITY): Protocol for a Randomized Controlled Trial

**DOI:** 10.2196/58580

**Published:** 2024-08-08

**Authors:** Elizabeth Mollard, Deirdre Cooper Owens, Christina Bach, Cydney Gaines, Shannon Maloney, Tiffany Moore, Christopher Wichman, Neel Shah, Michele Balas

**Affiliations:** 1 College of Nursing University of Nebraska Medical Center Omaha, NE United States; 2 Departments of History and Africana Studies University of Connecticut Storrs, CT United States; 3 College of Public Health University of Nebraska Medical Center Omaha, NE United States; 4 Department of Biostatistics College of Public Health University of Nebraska Medical Center Omaha, NE United States; 5 Maven Clinic New York, NY United States

**Keywords:** maternal health, health disparities, doula, African American, mobile phone

## Abstract

**Background:**

Black women are significantly more likely to experience severe maternal morbidity and are 3 times as likely to die from pregnancy-related causes compared to White women. Using a strengths-based wellness approach within an integrated supportive care program provided by a community doula could offer pragmatic solutions for Black maternal disparities. The Protective Assets Reinforced with Integrated Care and Technology (PARITY) program consists of a wellness technology platform, including informational links to wellness content and reinforcing motivational SMS text messages, as well as community-based doula support delivered both in person and through the technology platform to improve Black maternal wellness.

**Objective:**

This pilot randomized controlled trial (RCT) and mixed methods evaluation aims to (1) determine the feasibility and acceptability of the PARITY intervention; (2) investigate the preliminary efficacy of the PARITY intervention on clinical outcomes (maternal blood pressure, gestational weight gain, and cesarean birth); and (3) investigate changes to wellness behavioral outcomes (nutrition, physical activity, sleep, and health care adherence) and empowered strengths (self-efficacy, social support, motivation, resilience, problem-solving, and self-regulation) in the intervention group compared to a control group.

**Methods:**

A 2-arm RCT and mixed methods evaluation will be conducted. Overall, 60 Black pregnant individuals will be randomized in a ratio of 1:1 to either the intervention or informational control group. Participants in the intervention group will receive access to the technology platform over a 12-week period that ends before birth. Intervention participants will be assigned a doula interventionist, who will meet with them 4 times during the intervention. All participants (intervention and control) will receive a referral for a birth doula at no cost, printed materials about having a healthy pregnancy, and community resources. Feasibility and acceptability will be assessed at the end of the program. Measures will be obtained at baseline (20-28 weeks), the 36th week of pregnancy, birth, and 6-12 weeks post partum. Summary statistics and distribution plots will be used to describe measured variables at each time point. A generalized linear mixed model with a shared random component will be used to analyze the effects of PARITY on clinical, wellness behavioral, and empowered strength outcomes, including baseline nutrition, physical activity, and sleep measures as covariates. For significant effects, post hoc contrasts will be adjusted using the Holm method to maintain comparison-wise error at or <.05. Missing data will be addressed using a pattern-mixture model.

**Results:**

The National Institute of Nursing Research funded this pilot RCT. Recruitment, enrollment, and data collection are ongoing, and the estimated study completion date is October 2024.

**Conclusions:**

The expected results of this study will provide the feasibility and preliminary efficacy of the PARITY intervention, to be used in a larger trial with a 12-month PARITY program intervention.

**Trial Registration:**

ClinicalTrials.gov NCT05802615; https://clinicaltrials.gov/study/NCT05802615

**International Registered Report Identifier (IRRID):**

DERR1-10.2196/58580

## Introduction

### Background

The United States spends more on health care than any other nation in the world yet has the highest maternal morbidity and mortality rates of all high-income nations [1,2]. Severe maternal morbidity, defined by the Centers for Disease Control and Prevention as maternal outcomes that result in significant or life-threatening consequences for a woman’s health, has increased by approximately 200% over the past 20 years, while maternal mortality has increased by 37% since 2018 [3-5]. Black women experience higher rates of pregnancy-related complications and are more likely to die from pregnancy or childbirth compared to White women, regardless of comorbid conditions or socioeconomic status [6-9]. This gap has not narrowed in decades, exposing Black families and their communities to profound emotional, social, and economic consequences that ripple for generations [10]. The effects of increased severe maternal morbidity in Black communities are wide ranging and include higher health service use, higher direct medical costs, extended hospital stays, long-term rehabilitation, ongoing chronic health conditions, disability, and earlier death [11-13].

Most studies regarding susceptible maternal populations commence from a deficit-based discourse, which refers to communication that focuses on deficiencies, lacks, or failures, ultimately perpetuating biases and hindering efforts to address disparities [14,15]. To counteract this deficit-based discourse, it is important to examine, include, and emphasize the strengths and protective assets of Black maternal populations [16]. An asset-based or strengths-based approach uses the positives of an individual, such as their knowledge, skills, and community, as the basis to create protective processes in health and well-being [14]. This study is an example of research designed using an asset-based approach [14], intentionally including measures and tools that elucidate the many strengths of Black maternal populations. This research is framed by the concepts of empowerment and wellness, which are principles of the midwifery model of care [17]. The use of a strengths-based approach helps buffer the deficit discourse participants may have internalized through previous interactions with health care services; highlights the assets of participants; and gives them tools for wellness that can be leveraged in confronting, overcoming, and maintaining resilience to risks within their lives.

Community-integrated care is a solution that empowers Black women to be healthy within the context of the neighborhoods and communities in which they live [18]. Community health workers (CHWs) are trusted members of their community who facilitate access to services and improve health care quality by providing community education, social support, and advocacy [19,20]. In our previous work, an intervention using a prenatal technology platform with CHW reinforcement translated to cost savings on health care use averaging US $1079 for intervention participants compared to control participants [21]. Doulas are a type of CHWs who are trained support persons and advocates who work with individuals during the pregnancy continuum and are uniquely situated to help overcome maternal health disparities [22,23]. Doula care has been shown to improve women’s experiences; is associated with improved maternal outcomes; and helps mitigate the effects of social determinants of health through better health literacy, social support, and cultural competence [22].

The theory of maternal adaptive capacity is a strength-focused theoretical framework that focuses on the protective assets one may use to achieve or maintain health throughout pregnancy [24]. On the basis of the theory of maternal adaptive capacity and our preliminary work, this randomized controlled trial (RCT) and mixed methods evaluation examines a novel, strengths-based intervention for pregnant Black women [21,24]. This intervention is entitled the Protective Assets Reinforced With Integrated Care and Technology (PARITY) program and consists of a wellness technology platform and community-based doula support. PARITY is a strengths-focused intervention that uses mobile technology and doula support to empower individual women to bolster their existing strengths (protective assets) to navigate the health care system, improve overall wellness behaviors, and improve maternal outcomes.

### Objectives

The overall purpose of this PARITY program is to reinforce protective assets through empowering messaging, promote wellness behaviors, and support Black birthing persons. This pilot trial will provide information on the feasibility, acceptability, and preliminary efficacy of the PARITY intervention among Black pregnant women as well as provide critical information on study design, retention, effect sizes, and outcomes assessment.

The objectives of this study are to (1) explore the feasibility and acceptability of the PARITY intervention; (2) investigate the preliminary efficacy of the PARITY intervention on changes to maternal blood pressure (primary), gestational weight gain, and cesarean birth (secondary) and estimate the effect size of the intervention relative to the control group; and (3) investigate changes to wellness behavioral outcomes (nutrition, physical activity, sleep, and health care adherence) and empowered strengths (self-efficacy, social support, motivation, resilience, problem-solving, and self-regulation) in PARITY intervention participants. With the combination of information, guidance, and support toward leveraging their innate strengths into actionable wellness, we hypothesize that the PARITY program will improve maternal blood pressure, gestational weight gain, and cesarean birth rate in the intervention group compared with the information-only control group. Further, we expect emphasizing individual strengths and building from them while navigating pregnancy-related health care will improve wellness behaviors and empowered strength variables such as adherence to medical care, including appointment adherence.

## Methods

### Study Design

This study is a 2-arm prospective RCT and mixed methods evaluation of the PARITY intervention for pregnant Black women. Participants will be randomized to either the information control group (30/60, 50%) or the intervention group (30/60, 50%) in a 1:1 ratio. A sample size of 30 participants per group provides 80% power to detect a significant difference at a .05 level while accounting for attrition [25-27]. Select intervention participants (10/60, 17% to 15/60, 50%) will engage in a qualitative interview at 12 weeks post partum to discuss their experiences, opinions, and suggestions for the intervention. Participants will be enrolled in the study for up to 24 weeks. This time frame will include enrollment, the intervention, and postpartum follow-up assessments. Due to the nature of the intervention, the participants, interventionists, and research assistants will not be blinded. However, standardized training and standard operating procedures, including the use of deidentified self-reported questionnaires administered at baseline and after the intervention period, will reduce the potential for bias due to not blinding.

### Study Population

Participants will be included in the study based on the following criteria: (1) self-identify as being of Black race, (2) pregnant with gestational age 20 to 28 weeks at enrollment, (3) ability to read and write in the English language, (4) planning to give birth in a health care facility and receive obstetrical care with a health care record, (5) reside within 20 minutes of the identified metropolitan area, (6) 19 to 51 years of age, and (7) own a smartphone with internet access. Participants will be excluded from the study for the following reasons: (1) current involvement in another intervention study, (2) planned pregnancy termination, or (3) inability to provide informed consent.

### Intervention

#### Overview

The PARITY program is designed to occur throughout the pregnancy continuum (first trimester through 12 months post partum). For the purposes of this study, the PARITY program will be shortened in length and scope to show feasibility for a larger trial. Participants will be enrolled in the study from 20 to 28 weeks’ gestation of pregnancy and will complete postintervention surveys at 6 to 12 weeks post partum. All participants (intervention and information control groups) will receive routine care from obstetric providers (not paid for by the study), printed materials about having a healthy pregnancy, printed materials about community resources, and printed materials with a referral to a birth doula if interested (paid for by the study as an incentive).

The PARITY program involves 2 parts. The first is a wellness technology platform that includes a wellness curriculum and reinforcing SMS motivational messages with links to in-depth wellness content. The second is Black community–based doula support, delivered both in-person and through the technology platform.

The mobile technology component of the intervention is a web-based platform hosted by GoMo Health delivered over a 12-week period and will end before birth [28]. The platform includes web pages with various pregnancy-related wellness content. The content is evidence based, tested by GoMo Health, and based on our preliminary research [21,28]. Participants will receive SMS *text* messages from the web platform, and at times these will include hyperlinks. The doula will be able to communicate with participants, reinforce its content, and hold the intervention sessions within this platform [28]. The platform was approved by the University of Nebraska Medical Center Information Technology Services and is compliant with the Health Insurance Portability and Accountability Act. Participants in the intervention group will receive 4 sessions with the doula interventionist within the technology platform and 12 weeks of tailored wellness, strengths, and health care adherence curriculum and SMS text messaging through the platform. There is a comprehensive database to track calls, opened hyperlinks, and participant engagement with the platform.

#### Components of the Intervention

##### Strengths

Participants will receive 12 strengths-focused SMS text messages sent through the web-based technology platform. They will also receive 4 wellness messages with hyperlinks to more in-depth educational content within the web platform related to nutrition, physical activity, and sleep. The content of the messages for this study will be tailored to maternal wellness in Black women and informed by focus groups comprising Black community members [29]. In total, participants will receive 24 SMS text messages over 12 weeks. Messages will focus on improving self-perception, reminding women they can make positive changes and be healthy, instilling a sense of optimism for the future, and imparting competence regarding challenges that may be faced throughout pregnancy and beyond. In addition, messages will convey how strengths can provide buffers against adversity and promote resilience to risk. Messages that promote positive feelings, such as gratitude, humor, and hope, will also be reinforced through a strengths-focused framework.

##### Nutrition

PARITY includes information on nutrition during pregnancy and beyond. Nutritional advice will focus on eating foods in unprocessed forms (eg, fruits, vegetables, and lean meat), preparing food instead of purchasing fast food, mindfulness in eating, and water consumption. These nutritional guidelines are consistent with healthy weight gain and multiple nutritional benefits without prescribing standards that may require excessive financial resources or exclude foods that may be culturally important to participants.

##### Physical Activity

PARITY includes approaches to increase physical activity and reduce sedentary behaviors. The goal is to significantly increase each individual’s physical activity level above baseline. Emphasis will be placed on approaches to exercise safely in pregnancy, finding joy in body movement, walking, and easy ways to reduce sedentary behavior in daily life (eg, using the stairs instead of the elevator).

##### Sleep

The sleep component of the PARITY intervention addresses ways to improve the quality and duration of sleep in pregnancy and beyond. The content will focus on the benefits of sleep to wellness and pregnancy, sleep hygiene, and troubleshooting sleep problems. Strategies, such as relaxation techniques, regularizing the sleep schedule, stimulus control, and adjusting the bedtime will be included. Specific strategies for improving sleep duration and quality during pregnancy related to nighttime waking and nocturia will also be discussed.

#### Health Care Adherence

Health care adherence content will include general reminders about adhering to the patient’s individually prescribed health care plan and reminders of appointments. The content will also include information on how to approach health care providers with questions and concerns and the importance of engaging with the medical system despite current or past negative experiences and resources on accessing care if needed.

#### Doula Sessions

Each participant will work with a Black community–based doula interventionist who will communicate with them via the PARITY mobile technology platform during the study. The doula interventionist will have certifications as a doula and training in emotional and educational support, pregnancy health, privacy and confidentiality, inspiring change (theories of health), various learning styles, health literacy, and cultural competency. All doula sessions will be conducted by the same doula interventionist, who will be blinded to all outcome data.

The SMS text messaging will be sent in the doula’s voice. The doula interventionist will communicate with each participant in 4 live sessions lasting 30 to 60 minutes. These live conversations will include basic instruction and open-ended conversations focused on reinforcing strengths and wellness habits. In addition, these check-ins will promote health care adherence and provide guidance on navigating the health care system. The doula interventionists will assist participants through the strengths-focused framework of maternal adaptive capacity to identify and understand each participant’s particular barriers to wellness and health care adherence and to design approaches that increase behavioral change [24]. The doula interventionist will suggest community resources based on the participant’s needs.

#### Qualitative Interviews

The qualitative interview uses the strengths-focused framework of the theory of maternal adaptive capacity to better understand the acceptability of the intervention and its role in emphasizing protective assets in Black pregnant women [24]. A total of 10 to 15 participants from the intervention group will be interviewed at 12 weeks post partum. A semistructured interview guide will be used to gather in-depth qualitative information. The interview guide consists of open-ended questions and additional probes that explore participants’ experiences and opinions regarding the various intervention components. The interviewer will have the flexibility to ask additional questions for further clarification and to ensure comprehensive understanding.

### Information Control Group

Participants assigned to the information control group will receive routine care from obstetric providers (not paid for by study), printed materials about having a healthy pregnancy, printed materials about community resources, and printed materials with a referral to a birth doula if interested (paid for by the study and offered as an incentive).

### Outcomes

The feasibility of a full trial will be monitored throughout the study (eg, the randomization, recruitment, and retention of participants; data collection; and adherence). Additional data will be collected only on intervention participants. These include data on engagement with the PARITY intervention based on the number of hyperlinks clicked, time engaged in the content, and the number of doula sessions completed throughout the 12 weeks. A total of 10 to 15 intervention participants will be asked to complete a qualitative interview to discuss their experiences with the intervention and the acceptability of the technology component, doula component, and strength messaging component at 12 weeks post partum.

Clinical maternal outcomes (maternal blood pressure, gestational weight gain, delivery modality, and exploratory perinatal outcomes) will be measured at 20 weeks, 36 weeks, birth, and 6 weeks post partum and will be collected from the medical record. At baseline (20-28 weeks’ gestation), demographics, discrimination, and neighborhood variables will be assessed using self-report questionnaires administered via REDCap (Research Electronic Data Capture; version 14.3.11) [30,31]. At baseline and 6 to 12 weeks post partum, wellness-related behavior outcomes (nutritional intake, physical activity, and sleep quality) and empowered strengths (self-efficacy, social support, problem-solving, motivation, resilience, and self-regulation) will be measured using web-based self-report questionnaires in REDCap. In addition, health care adherence (the number of prenatal appointments) will be evaluated retrospectively using medical records.

### Recruitment

Prospective participants (intervention and control) will be recruited at the beginning of their pregnancy up to 28 weeks of gestation about interest and participation in the study. Participants will be recruited via advertisements on social media, fliers, and word of mouth. If an individual is interested in participating, they will contact the research team by completing the study screening questionnaire in REDCap or via telephone or email. Study personnel will contact the potential participant via telephone or email and explain the study in detail. Once interest is determined and screening eligibility is met, participants will be invited to sign a consent form and a medical release of information form and complete the baseline assessments at 20 to 28 weeks of pregnancy.

### Participant Allocation

Randomization allocation will be guided by the National Cancer Institute Clinical Trial Randomization Tool [32]. This tool generates an allocation scheme using the asymptotic maximal procedure. The following parameter inputs were used in the tool: trial size=60, maximum-tolerated imbalance=3; and arms (ratio 1:1). The output of this randomization allocation tool provides a schedule of randomization.

After signing the informed consent and release of medical record forms, participants will be emailed a secure link to complete the baseline enrollment surveys electronically in REDCap. In addition, each participant will be given the Healthy Pregnancy handout [33], Community Resources handout, and Birth Doula handout, in addition to the standard consent documents. Participants will be randomized according to the randomization schedule based on when they complete the enrollment surveys. Once a participant completes the enrollment surveys, the research coordinator will inform the participant of their group assignment and explain the procedures related to the assigned group.

Individuals randomized to the information control group will be dismissed and informed that they will be contacted by study personnel 6 to 12 weeks post partum to complete the follow-up assessments. Individuals randomized to the intervention group will be contacted by the doula interventionist to schedule their first doula session and be onboarded to the PARITY platform. The interventionist will explain that the participant will receive 3 additional sessions (4 in total) with the doula interventionist in addition to the mobile technology platform where communication can occur with the doula.

During the sessions, the doula interventionist will use the Doula Sessions Manual, which contains a script for each visit. The doula interventionist will track when contact is made with the participant on a form called the Intervention Fidelity Checklist. To ensure the fidelity of the intervention, the doula interventionist’s adherence to the intervention protocol will be measured through an audit of the dates and missed visits on the Intervention Fidelity Checklist and monthly review meetings.

All participants will complete postintervention surveys at 6 to 12 weeks post partum. At approximately 12 weeks post partum (or upon the receipt of records), participant medical records will be accessed to extract the pertinent outcome data. A total of 10 to 15 participants in the intervention group will be approached about completing the qualitative portion of the study. Participants will be approached in order based on the study’s completion date.

### Ethical Considerations

This protocol was approved by the University of Nebraska Medical Center Institutional Review Board (IRB; #0076-23-EP). Participants must sign an informed consent form before participating in the study. The consent process involves a detailed description of the study, describing any participation risks, indicating that participation is voluntary and that the participant has the right to withdraw at any time. Informed consent will be obtained in a private, quiet location to facilitate discussion and thoughtful consideration. Research team members will assess participants’ capacity to consent, answer questions, and ensure understanding.

To maintain participant confidentiality, all study data will be deidentified. Each participant will be assigned an identification code for use on all study materials. The link between a participant’s name and identification code will be stored only on the consent form, which will be kept in a locked file in the principal investigator’s office, separate from other study materials. Data sources include consent forms; medical records; transcribed interviews, handwritten notes; and data entered in REDCap, a secure web-based application for research data management [30,31]. Hard copies of any data will be stored in a locked file accessible only to the investigators or the IRB. All electronic data, including entries in REDCap, will be password protected and encrypted. Medical record data will be encrypted and transmitted through secure firewalls. All transcripts from the qualitative interviews will be deidentified, encrypted, stored in a locked facility, and available for analysis and review only by study staff. Only study personnel, the research sponsor, the IRB, and legally authorized individuals will have access to the research records. Upon study completion, all personal health information will be removed from the database. Study findings will be published in scientific journals or presented at meetings without revealing patient identities.

Participants will receive a US $50 gift card for completing baseline assessments and US $50 for completing follow-up assessments. Participants in the qualitative portion of the study will be compensated with a gift card valued at US $25.

### Outcome Measures

At enrollment, participants will sign a medical release of information form allowing the research team to measure data retrospectively from the participant’s medical records. To collect the self-report questionnaires for the enrollment and postpartum assessments, each participant will use a unique, secure link to complete the electronic questionnaires in REDCap at baseline after informed consent is signed and at 6 to 12 weeks post partum based on the participant’s due date. Reminders from the research team will be sent to participants using various forms of contact (phone and email) to complete the questionnaires as needed.

The following outcome measures will be used to examine the feasibility and acceptability of the PARITY intervention:

1. Feasibility will be assessed through enrollment, attrition, adherence to the intervention, and data collection.
  - Enrollment rate: enrollment will be measured by the percentage of eligible individuals who agree to participate in the trial.
  - Attrition rate: attrition will be measured by the percentage of eligible individuals who complete the study.
  - Adherence to the intervention: adherence to the intervention will be measured using the Intervention Fidelity Checklist, where the doula interventionist will track when contact is made with participants and the number of doula sessions completed. Engagement with the platform will be tracked through clicks and the time spent on the platform.
  - Data collection: data collection feasibility will be measured through study personnel’s sense of ease of use, time required, and missing data.
2. Acceptability of and experiences with the intervention will be assessed using in-depth qualitative information gathered through interviews with intervention participants at 6 to 12 weeks post partum. Interviews will solicit (1) the usefulness of the intervention, (2) ideas to improve the intervention, (3) satisfaction with the intervention, and (4) the utility of innovative technology to deliver the intervention. The qualitative interviews will be audio recorded and transcribed verbatim.

The following outcome measures will be used to examine the preliminary efficacy of the PARITY intervention:

1. Maternal blood pressure (primary) at 20 weeks, 36 weeks, birth, and 6 weeks post partum will be measured retrospectively from the medical record at 12 weeks post partum. The data will be analyzed as a continuous variable using the participant’s blood pressure (systolic and diastolic) as well as categorically with the blood pressure classifications of hypertension (>140/90) and severe hypertension (>160/110).
2. Gestational weight gain (secondary), or the amount of weight gained in pounds at 20 weeks, birth, and 6 weeks post partum, will be measured retrospectively from the medical record at 12 weeks post partum.
3. Cesarean birth (secondary) will be assessed from the delivery modality (vaginal, cesarean, or assisted vaginal delivery), measured retrospectively from the medical record at 12 weeks post partum.
4. Perinatal outcomes (exploratory) from birth will be measured retrospectively at 12 weeks post partum via the maternal medical record. The perinatal outcomes that will be measured include preterm delivery; gestational diabetes; birth weight; gestational age at delivery; anesthesia used during labor; labor length; delivery provider type (obstetric physician, family physician, resident physician, or certified nurse midwife); and intrapartum medical diagnoses according to the
  International Classification of Diseases, 10th Revision
  [34], including hypertensive disorders of pregnancy, intrapartum fever, and postpartum hemorrhage. In addition, the Appearance, Pulse, Grimace response, Activity, and Respiration score of the newborn at birth will be measured as a way to summarize the health of the baby [35].

The following outcome measures will be used to investigate changes to wellness behavioral outcomes and empowered strengths:

1. Wellness-related behavioral outcomes:
  - *Nutritional intake* will be measured with the Multifactor Screener, 2000, as used in the Observing Protein and Energy Nutrition Study [36]. This instrument measures approximate intakes of fruits and vegetables and percentage energy from fat and fiber. Respondents will report through a self-administered questionnaire in REDCap how frequently they consume foods in 16 categories and the type of milk consumed. The questionnaire will measure nutrition at baseline and 6 to 12 weeks post partum.
  - *Physical activity* will be measured with the International Physical Activity Questionnaire-Short Form (IPAQ-SF) [37]. The IPAQ-SF consists of 9 items and collects information on the time spent walking, in vigorous and moderate intensity activity, and in sedentary activity over the last 7 days. It is scored by converting the reported time into metabolic equivalent of task (MET) minutes. The MET minutes are then summed to provide an understanding of an individual’s physical activity levels, with low levels being <600 MET minutes per week, moderate activity levels being 600 to 2999 MET minutes per week, and high activity levels being ≥3000 MET minutes per week [37]. It will be self-administered in REDCap and takes approximately 10 minutes to complete. The IPAQ-SF is a reliable (α>.80) and valid instrument [37]. Physical activity will be measured at baseline and 6 to 12 weeks post partum.
  - *Sleep quality* will be measured using the Pittsburgh Sleep Quality Index, a reliable (α>.83) and valid self-report questionnaire that assesses sleep quality over a 1-month time interval [38]. The measure consists of 19 individual items, creating 7 component scores that range from 0 to 3 points. The 7 component scores are summed to produce 1 global score that ranges from 0 to 21 points, with 0 indicating no difficulty and 21 indicating severe difficulty across all areas [38]. Sleep quality will be measured through a self-administered survey, which takes about 15 minutes to complete, in REDCap at baseline and 6 to 12 weeks post partum.
  - *Health care adherence* will be measured by the number of health care appointments attended and missed, which will be logged in the medical record. Health care adherence will be measured throughout the pregnancy continuum and collected at 12 weeks post partum retrospectively via the medical record.
2. Empowered strengths:
  - *Self-efficacy* will be measured using the General Self-Efficacy scale [39] administered in REDCap. The General Self-Efficacy scale is a valid and reliable (α>.75-.90) 1D self-report measure that features a 10-item questionnaire assessing the optimistic self-beliefs of individuals to cope with various challenging demands in life [39]. It takes approximately 5 minutes to complete. The response options are presented along a Likert scale for each item, ranging from 1 (not at all true) to 4 (exactly true). The total score is calculated as the sum of the items, with a higher total score indicating more self-efficacy [39]. Self-efficacy will be measured at baseline and 6 to 12 weeks post partum.
  - *Social support* will be measured using the 10-item Social Provisions Scale [40]. The 10-item Social Provisions Scale is a validated and reliable (α>.80) measure that assesses 5 social provisions: attachment, guidance, social integration, reliable alliance, and reassurance of worth [40]. Each item is rated on a 4-point Likert scale, ranging from 1 (strongly disagree) to 4 (strongly agree), and the individual item scores are then summed to provide a summary score ranging from 10 to 40 [40]. Higher scores indicate higher levels of social support [41]. Social Support will be measured through a self-administered survey, which takes approximately 5 to 10 minutes to complete, in REDCap at baseline and 6 to 12 weeks post partum.
  - *Motivation* will be measured with the Self-Motivation Inventory (SMI) [42]. The SMI is a 40-item, 5-point Likert scale questionnaire that will be self-administered in REDCap and takes about 15 minutes to complete. The SMI is a valid and reliable (α>.91) instrument measuring self-motivation conceptualized as perseverance independent of external support [42]. Motivation will be measured at baseline and 6 to 12 weeks post partum.
  - *Resilience* will be measured using the 10-item Connor-Davidson Resilience Scale [43]. The 10-item Connor-Davidson Resilience Scale is a valid and reliable (α>.85) unidimensional self-reported scale measuring resilience [43]. Respondents will self-report through a survey in REDCap by rating items on a 5-point Likert scale ranging from 0 (not true at all) to 4 (nearly all of the time). Scores range from 0 to 40, with higher scores representing higher resilience [43]. Resilience will be measured at baseline and 6 to 12 weeks post partum through the survey, which takes about 5 minutes to complete.
  - *Problem-solving* will be measured with the Problem-Solving Inventory (PSI) [44]. The PSI is a valid and reliable (α>.86) self-report instrument that measures how well individuals make decisions and their problem-solving abilities [44]. The PSI includes 32 items using a 6-point Likert scale and takes about 10 minutes to complete. The total score of the questionnaire is the sum of all responses, with lower scores representing lower problem-solving abilities [44]. Problem-solving will be measured through a self-administered survey in REDCap at baseline and 6 to 12 weeks post partum.
  - *Self-regulation* will be measured with the Index of Self-Regulation (ISR) [45]. The ISR is a valid and reliable (α>.87) instrument that measures the level of self-regulation for health behavior change in a 9-item scale [45]. Respondents rate items on a 5-point Likert scale, ranging from 1 (strongly disagree) to 6 (strongly agree), with higher sum scores indicating higher levels of self-regulation for a behavior [46]. Self-regulation will be measured at baseline and 6 to 23 weeks post partum in REDCap through the ISR, which takes about 5 to 10 minutes to complete [46].
3. Other variables that will be assessed are demographics, discrimination, and neighborhood:
  - *Demographics:* an investigator-developed form will measure demographic variables, including income, education, ethnicity, insurance, health provider, relationship status, and pregnancy history, at baseline.
  - *Discrimination:* discrimination will be measured using the Everyday Discrimination Scale [47] through a self-administered survey in REDCap. The Everyday Discrimination Scale is a valid and reliable (α>.74) instrument that measures the frequency of chronic and routine unfair treatment in everyday life [47]. Respondents are asked to report how often they experience unfair treatment in their day-to-day life on a 6-point Likert scale ranging from 1 (never) to 6 (almost every day), with total scores ranging from 10 to 60. Higher scores indicate experiencing discrimination frequently and in a variety of situations [47]. Discrimination will be measured at baseline.
  - *Neighborhood:* neighborhood variables will be assessed using the Neighborhood Collective Efficacy-Community Cohesion and Informal Social Control protocol from the Project on Human Development in Chicago Neighborhoods [48]. This valid and reliable (α=.82) measure includes 10 Likert-style questions that assess trust and expectations among neighbors and is used to determine how the neighborhood environment modifies risky behaviors [48]. It will be self-administered as a survey in REDCap and takes about 10 minutes to complete. The measures consist of 2 subscales, Community Cohesion and Informal Social Control, that include 5 Likert-style items, each ranging from 1 (strongly disagree) to 5 (strongly agree). Higher scores indicate greater neighborhood collective efficacy [48]. Neighborhood variables will be measured at baseline.

### Data Analysis

Participants will be described using standard summary statistics and distribution plots of measured variables at each point in time. We will estimate the standardized magnitude of each baseline difference and the correlation between each baseline variable and each outcome of interest in the interim and primary end point of early participants to objectively determine baseline variables that will be needed to estimate the unbiased effect of PARITY on each outcome. A shared random component generalized linear mixed model (GLMM) will be used to simultaneously describe and compare the effects of PARITY on clinical outcomes, wellness-related behavioral outcomes, and empowered strength outcomes over the measurement periods [49]. A shared random component model will account for the correlation within participants on any 1 measure and the correlation between measures on the same participant at the same time point. The effect of time will be treated as a fixed effect. The time-by-group interaction will be tested; if the interaction *P* value is >.10, it will be dropped from the model. Baseline nutrition, physical activity, and sleep measures will be used as covariates to control for potential differences in the groups and to avoid detecting spurious time-by-treatment interactions due to the random assignment of participants to groups. Demographic or clinical characteristics exhibiting differences between groups at baseline will be investigated as potential covariates in the models. For significant effects, preplanned post hoc contrasts will be computed, and *P* values will be adjusted using the Holm method to maintain comparison-wise error at or <.05 [50]. Unless otherwise specified, the significance level for all tests will be.05.

Missing outcome data caused by loss of follow-up are possibly related to the participant’s unobserved outcome measures (nonignorable missingness). The GLMM provides unbiased estimates under missing completely at random and missing at random mechanisms. The data will be reanalyzed using a pattern-mixture model (PMM) to allow for different coefficient changes based on dropout patterns [51]. GLMM and PMM marginal treatment effects will be compared. If the marginal treatment effects differ by >10%, the missingness mechanism will be assumed not at random, and the PMM model marginal estimates will be used to draw conclusions.

Interview data will be analyzed using a thematic analysis approach [52]. The audio recordings will be transcribed verbatim, and the accuracy of the transcripts will be verified by a separate team member who will cross-reference the transcriptions with the audio recordings. Transcripts from interviews will be independently coded in a word processing software by 2 members of the research team. Coding will be completed using an inductive approach to look for specific to more broad patterns across the interview data collected [53]. This will allow for themes and patterns to emerge directly from the data. A list of codes will be recorded and described in a codebook along with the definitions and descriptions of codes and representative quotes from the data so that codes have distinct boundaries. After the data are independently coded, the 2 researchers will discuss the codebook and resolve any discrepancies. The codebook will also help to assess interrater reliability among team members in applying codes to the data [54]. Codes will then be categorized into potential themes by clustering similar codes from participant responses. Audio recordings will be destroyed after transcription has been confirmed. Transcriptions will be destroyed at the end of the study.

### Sample Size Calculation for Future Full-Scale RCT

A sample size of 25 participants per group is sufficient to measure the proportional outcomes of recruitment efficiency and attrition for each group separately with an SE ≤0.10, assuming no attrition and worst-case variability (0.25) in each group. Past research suggests that attrition of up to 20% is acceptable and limits bias [26,27]. If there is up to 20% attrition in each group, the SE for each proportional outcome will be ≤0.12.

On the basis of Welch 2-sample 2-tailed *t* test, a minimum sample size of 25 participants per group provides 80% power to measure a difference in mean blood pressure and gestational weight change between the intervention and control groups of 0.81 multiplied by the SD, using a significance level of .05 [25]. If there is up to 20% attrition in each group, a difference of 0.91 multiplied by the SD is detectable with 80% power using a significance level of.05 [25]. We will recruit at least 5 additional participants per group, planning for screen failures or participant withdrawals.

## Results

This pilot RCT is funded by the National Institute of Nursing Research. Data collection was initiated in August 2023 and will continue for approximately 2 years. We will report on the outcomes identified above in a peer-reviewed journal. We anticipate that the results of this feasibility RCT will inform a larger RCT with a 12-month version of PARITY.

## Discussion

### Overview

This pilot RCT and mixed methods evaluation will provide information on the feasibility, acceptability, and preliminary efficacy of the PARITY intervention in Black pregnant women, as well as provide critical information on study design, retention, effect sizes, and outcomes assessment. We anticipate finding improvements in maternal blood pressure, gestational weight gain, and cesarean birth rate in the PARITY program intervention group compared with the information-only control group. In addition, we expect that focusing on strengths and leveraging them during pregnancy will improve wellness behaviors and empowered strength variables, such as adherence to medical care and appointment compliance.

Previous work seeking to understand the mechanisms of Black maternal health disparities have characterized the negative outcomes experienced by Black women as socioeconomic in nature [55,56]. While these factors are associated with maternal morbidity and mortality, Black maternal health disparities cannot be explained entirely by factors such as income, educational level, and health insurance status [7,9]. Further, this narrative is problematic, as it reinforces the stereotype that Black women have low income and are undereducated [57,58]. Indeed, our previous work involving nearly 10,000 nulliparous mothers showed that Black women with higher education and adequate income were more likely to experience maternal morbidity than their counterparts of other racial or ethnic backgrounds who had lower education and income levels [59]. A growing body of evidence suggests that disparities in Black maternal health outcomes are not rooted in biology. Instead, discrimination and bias due to race are driving contributors to the decreased rates of prenatal care, reduced quality of care, and poor health outcomes for childbearing women of color [60-62]. The failure of prior research to ameliorate maternal health disparities may be related to the prevailing deficit-based framework through which maternal health research is designed and disseminated [9].

### Strengths

This research study uses the following strategies to work on overcoming deficit discourse through a strengths orientation: (1) includes a focus on capabilities, strengths, and opportunities; (2) conducts research with and by instead of about the individuals or groups; (3) promotes hope by using language that is person centered and optimistic and leads to positive perceptions; (4) identifies social context and the resources that exist within communities; (5) includes research questions that lead to empowerment; and (6) engages research participants with respect, allowing opportunities in which they offer their expertise about their situation [16].

In addition, current maternal health research rarely focuses on how Black women can become or remain well. Along with health care adherence, wellness includes daily lifestyle habits, such as physical activity, nutrition, sleep, and connectedness, that promote optimal physical and mental health. Our previous work showed that simple wellness-related lifestyle changes, such as physical activity, improved nutrition, and better sleep, were associated with decreased pregnancy complications [52]. Despite being largely ignored in the severe maternal morbidity research literature, wellness strongly impacts maternal outcomes. Daily wellness habits focused on physical activity, nutrition, and sleep are easier for pregnant women to implement compared to complex interventions and have the potential to increase the lifelong health of women and their families. This intervention is designed to work on building strong daily wellness habits and to empower women with the knowledge and tools to make healthy choices.

This research offers a comprehensive, innovative approach to improving maternal wellness by integrating a strengths-based framework, novel technology, and peer-based community support while addressing access to care, quality of care, and each participant’s social needs and strengths. This research has the potential to provide pragmatic solutions for Black maternal disparities and shift the narrative about Black women in the research literature. Knowledge gained from this research may inform future research and practices related to community-engaged approaches and interventions.

### Challenges and Limitations

Due to the nature of pregnancy and the inexact times during which birth occurs, we expect that participants will receive varying doses of the intervention. This is a limitation of pregnancy-related research and will be accounted for in analyses. In addition, limitations on funding make it so that we cannot translate materials into other languages or provide translators for this feasibility study, limiting the population to English-speaking individuals. The intervention evaluated in this study requires participants to own a smartphone, which also limits the population that can be reached in this study. Finally, several measures obtained at the 4 main data collection time points might burden participants or lend themselves to missing data.

### Conclusions

Integrating a unique technology intervention alongside doula support represents a novel approach to maternal health interventions. This research will inform the feasibility of conducting a larger study and other future research involving community-engaged approaches. Moreover, the results hold promise in combating deficit discourse surrounding Black maternal populations, both within academic and clinical settings. By focusing on empowered wellness and leveraging strengths, this intervention aims to reduce maternal health disparities in Black women. The anticipated outcomes of this study will shed light on the effects of the PARITY intervention in addressing these disparities and may illuminate the role of individual protective assets in buffering against maternal health inequities.

## Appendix

Multimedia Appendix 1 Peer review report.

## Abbreviations

CHW: community health worker
GLMM: generalized linear mixed model
IPAQ-SF: International Physical Activity Questionnaire-Short Form
IRB: Institutional Review Board
ISR: Index of Self-Regulation
MET: metabolic equivalent of task
PARITY: Protective Assets Reinforced With Integrated Care and Technology
PMM: pattern-mixture model
PSI: Problem-Solving Inventory
RCT: randomized controlled trial
REDCap: Research Electronic Data Capture
SMI: Self-Motivation Inventory

## Data Availability

All data generated or analyzed during this study will be available in supplementary files or described directly in the main findings paper at the time of publication.
