# Supplementary material for: Protective Assets Reinforced With Integrated Care and Technology (PARITY): Protocol for a Randomized Controlled Trial
Source: JMIR Res Protoc. 2024 Aug 8;13:e58580. doi: 10.2196/58580 (PMC11342005; doi:10.2196/58580)
Supplement: Multimedia Appendix 1 [file resprot_v13i1e58580_app1.pdf]

MOLLARD, E

Modifications were made to proposal based on feedback and the partial funding that was offered. This reduced the scope of the project to a feasibility trial. Areas are redactated for confidential information.

[redacted]

[redacted]

CRITIQUE 1

[redacted]

Overall Impact: An application from an Early Career Scientist that proposes to determine the effects of the PARITY intervention on maternal morbidity, mortality, and health outcomes, assess mediation effects, and identify barriers, facilitators, and benefits of PARITY. To conduct this study, they are leveraging community partnerships (black doulas), community strengths, and technology to address the aims of improving maternal wellness of black women. This study is of high significance due to the high maternal morbidity and mortality rate of black women. Major strengths of the study include the use of doulas, mobile technology, and community partners. Although the team has experience with these topics, they do not have a track record or working together. Issues that dampened enthusiasm are related to lack of feasibility data for the team utilizing this concept in this population of women. Furthermore, lack of details in the approach related to technology support, specific surveys for outcomes dampen the enthusiasm for the successful completion and impactful outcomes of this proposal. Overall, this study has moderate impact.

1. Significance:

Strengths

- This project will focus on the strengths of black communities and individuals to help attenuate racial disparities in maternal morbidity and mortality, cesarean deliveries, nutrition, PA, sleep, healthcare adherence, mediation factors.

- Uses Doula (peer-based) support across 12-months and novel healthcare integrated mobile technology to address this important public health disparity among black pregnant women.

Weaknesses

- Although the topic is of high public import, the use doulas and community partners, and focusing on assets has been highlighted, it is not clear that the investigators have effectively utilized this approach individually or altogether to address these outcomes. Thus, it is not clear if the team is able to successfully implement these "tools" and to have measurable outcomes. (moderate-major)

2. Investigator(s):

Strengths

- [redacted]

Weaknesses

- [redacted]

3. Innovation:

Strengths

- The project aims to focus on the strengths of black individuals and communities in order to decrease maternal morbidity and mortality.
- Assess the program (use of Black doulas, mobile technology) on maternal morbidity/mortality, and wellness outcomes (nutrition, PA, sleep, healthcare adherence), identify mediators of outcomes, and identify barriers, facilitators, benefits of PARITY intervention.

- Although the use of technology itself is not novel, the application of this specific technology for this population is unique and warrants investigation.
- Wellness curriculum is an important addition to help address education and reinforcing healthy habits.

Weaknesses

- The assessment of maternal health outcomes is not novel for this population.

4. Approach:

Strengths

- The PARITY program will use standard prenatal care, doula care, mobile platform with messaging, and a wellness curriculum.
- There is great enthusiasm around the use of the doula sessions, wellness curriculum, and mobile technology as a timely means to interact with an underserved population.
- The EHR will use 21 CDC indicators for a composite severe maternal morbidity (SMM) score, delivery mode.

Weaknesses

- It is unclear internet service be considered an inclusion/exclusion criteria (moderate).
- It is unclear how the health (health, behavior, psychosocial) outcomes will be assessed
- The topic of surveys for nutrition, PA, and sleep quality is underdeveloped and unclear if current validated surveys will be used. (moderate for completion of Aim 4)
- There is no discussion regarding study attrition due to poor outcomes: pregnancy loss, postpartum conditions (mild-moderate).
- Concerns about the sample size and the proposal to decrease SMM from 15% to 5%

5. Environment:

Strengths

- The environment at UNMC is clearly supportive and sufficient for the study and is supportive of faculty researchers.

Weaknesses

- It is not clear if there is technical support for use/modification of the mobile technology as well as support for research participants.

Study Timeline:

Strengths

- Will recruit 384 participants across numerous year.

Weaknesses

- Year 1 refers to “Form a diverse transdisciplinary team,” but that should already be done.

- It is not known the difference between “Engage community to inform intervention” in Year 1 and “Engage diverse community” in Year 2, especially considering the diverse partners have been described in the application.
- It is unclear why diverse students are being recruited in Year 1, 3, and 5.
- It is not clear why stakeholders to identify barriers, facilitators, and benefits are being recruited in Year 5.

Protections for Human Subjects:

Acceptable Risks and/or Adequate Protections

Data and Safety Monitoring Plan (Applicable for Clinical Trials Only):

Acceptable

- o this should cover those who mention suicide or harm to the baby

Inclusion Plans:

- Sex/Gender: Distribution justified scientifically
- Race/Ethnicity: Distribution justified scientifically
- For NIH-Defined Phase III trials, Plans for valid design and analysis:
- Inclusion/Exclusion Based on Age: Distribution not justified scientifically
- what about those which have suicidal comments or talk about harm to the baby, how will this be handled?
- It is unclear why they are recruiting 12 year olds up to 51 years olds. Outcomes will be varied based on this wide range of age and the different physiological needs of the female body as well as social, societal differences.
- Recruitment table is for 384, but 454 in another part of the application; there needs to be cohesion related to the sample needed and justified to address this question.

Vertebrate Animals:

Not Applicable (No Vertebrate Animals)

Biohazards:

Not Applicable (No Biohazards)

Resubmission:

- N/A

Renewal:

- N/A

Revision:

- N/A

Resource Sharing Plans:
Acceptable

Budget and Period of Support:
Budget Modifications Recommended (in amount/time)
Recommended budget modifications or possible overlap identified:

- [redacted]

CRITIQUE 2

[redacted]

Overall Impact: This is a well written proposal from an early investigator with strong connections to the problem of disparities in maternal health and targeting their reduction. The conceptual framework of a strengths and resilience based view of black women and their community is novel and a critical means of looking at potential interventions. CHWs are widely used globally with excellent benefits for vulnerable populations but are understudied in the US. This strategy for peer and lay health worker interventions deserves greater study and the proposed work has much to commend it. The linkage of an mHealth technology platform with the on the ground work of a CHW serving as a Doula is also understudied in the US setting. Some limitations that reduced my enthusiasm include an inexperienced PI without a strong support system such as an MPI structure, the lack of a coherent linkage of such a support system with outcomes such as pre eclampsia which are mediated by physiologic changes that in themselves cannot be prevented but actually more likely to be detected by the involvement of a support system.

1. Significance:
Strengths

- Targeting the high risk low income Black population
- Taking on a novel strengths based approach (in contrast to the standard deficit model)
- Studying the use of Community Health Workers which are widely used globally but less so in the US
- Takling severe morbidity and mortality in this population

Weaknesses

- The linkage of strengths to outcomes is difficult to specify adequately to measure mechanisms – particularly when outcomes are disorders such as pre eclampsia which are likely to be detected earlier and more often with a support system such as this

2. Investigator(s):

Strengths

- Well positioned early investigator with commitment to this area of research
- The investigator team includes those appropriately trained and experienced to carry out this study (Balas)

Weaknesses

- The PI has not had NIH grant funding previously and there is significant risk that a project of this scale will have difficulties without a stronger support system

3. Innovation:

Strengths

- The use of a strengths rather than deficiency based model is innovative
- Linking a technology platform to the CHW/Doula intervention is innovative

Weaknesses

- None noted by reviewer.

4. Approach:

Strengths

- The individual randomization approach to the trial is robust and rigorous

Weaknesses

- Inadequate preliminary data linking the intervention elements to clinical outcomes
- Lack of a clear plausible linkage between the support intervention and the physiologic disorders identified as outcomes – likely those with support will have more disorders identified because they are being observed more
- The sample size will be underpowered to look at maternal mortality and to some extent severe morbidity, even in this high risk population

5. Environment:

Strengths

- Strong academic environments

Weaknesses

- The supports at the home institution are not as strong as they could be for this particular investigator, at their stage of development, and with this particular project – this adds some risk for the success of completing the study

Study Timeline:

Strengths

- Appropriate

Weaknesses

- None noted by reviewer.

Protections for Human Subjects:

Acceptable Risks and/or Adequate Protections

- Low risk to patients

Data and Safety Monitoring Plan (Applicable for Clinical Trials Only):

Acceptable

- Low risk study

Inclusion Plans:

- Sex/Gender: Distribution justified scientifically
- Race/Ethnicity: Distribution justified scientifically
- For NIH-Defined Phase III trials, Plans for valid design and analysis: Not applicable
- Inclusion/Exclusion Based on Age: Distribution justified scientifically

Vertebrate Animals:

Not Applicable (No Vertebrate Animals)

Biohazards:

Not Applicable (No Biohazards)

Resubmission:

- N/A

Renewal:

- N/A

Revision:

- N/A

Resource Sharing Plans:

Not Applicable (No Relevant Resources)

Budget and Period of Support:

Recommend as Requested

CRITIQUE 3

MOLLARD, E

[redacted]

Overall Impact: This is an R01 submission by a new investigator. This study proposes a randomized controlled trial of [redacted] Black pregnant women to test a pragmatic, community-centered solution to maternal health disparities that builds upon the inherent strengths of Black individuals and Black communities. The intervention is a previously developed program called Protective Assets Reinforced with Integrated care and TechnologY (PARITY), which includes community pairing with a doula and a mobile technology platform. Outcomes will include the effects of the PARITY Program on severe maternal morbidity (primary outcome), maternal mortality, and cesarean birth (secondary outcomes) in Black pregnant women; (2) Determine the effects of the PARITY program on wellness-related behaviors; (3) whether outcomes are mediated by changes in wellness behaviors; and (4) barriers, facilitators, and perceived benefits of the PARITY intervention. Overall, this is a very strong application with a high likelihood of significant scientific and health impact. The intervention is well designed and addresses key levers to improve maternal health. The study is well thought out and measures meaningful outcomes. The philosophical approach and frameworks used to guide the intervention and evaluation are appropriate (and developed by the PI). There are some minor limitations such as limited details on the Y1 efforts to create the technology platform, significant need for investing in training and hiring doulas, and stated primary reliance on social media for recruitment (although other recruitment mechanisms are presented in a table). My overall enthusiasm for this proposal remains high and this is a very well written, coherent, and strong proposal for a first time investigator.

1. Significance:

Strengths

- This is a well described and designed intervention that blends technology for self-care and personal contact for support and navigation. Collectively this has great potential for improving health outcomes.
- The proposed evaluation of the PARITY intervention is thorough, well thought out and complete. It will likely yield important data to understand the impact of PARITY as well as how to implement and spread it if effective.

Weaknesses

- The design costs and need for an expanded doula workforce may be difficult to sustain and disseminate, but if proven effective would create a justification for such investments.

2. Investigator(s):

Strengths

- The proposed transdisciplinary research team includes diverse and needed disciplines (nursing scientists, a Black historian, birth workers, a biostatistician, a public health scientist, and a physician medical advisor) and represents a variety of career stages (the PI is a first time investigator supported by more senior mentors) including students.
- Active inclusion of students from diverse and under-represented backgrounds is a strength.

- The inclusion of the Mallone Center and I Be Girl as community partners is a strength.
- There are concrete linkages between the timeline/milestones and the plan for enhancing diverse perspectives.
- The intervention grows from the Theory of Maternal Adaptive Capacity, a conceptual model developed and published by the PI.

Weaknesses

- No investigators have informatics expertise. To address this [redacted] is developing and delivering the mobile technology. There is limited description on their expertise or whether the team has worked with this group prior (I assume they have in their preliminary work).

3. Innovation:

Strengths

- The “great minds think differently” approach and the positive lens on protective assets are both strengths of this proposal.
- Linking technology and virtual care with personal real life care is likely necessary to impact outcomes.
- The pathway that doulas will provide integrated supportive care is well laid out in Table 1, spanning healthcare access, healthcare quality, and addressing social conditions.
- Group concept mapping is an innovative analytic approach.

Weaknesses

- None noted by reviewer

4. Approach:

Strengths

- The mixed methods evaluation around the four aims will nicely assess the impact of the proposed intervention and identify data to inform future implementation if it appears successful.
- Prior data on the PARITY prenatal technology platform seems promising, including more full term deliveries, more normal weight deliveries, and increase Patient Activation Measure scores.
- There is a well described and thorough plan to ensure the fidelity of the intervention and a process to measure the fidelity with which it is delivered and incorporate that into the analysis.
- Collecting data at four timepoints (baseline (16-20th week of pregnancy), 36th week of pregnancy, 12th week postpartum, and one year postpartum) will ensure a robust evaluation of the intervention impact. The outcomes shown in Table 3 are thorough, concrete, and span the desired domains to understand the impact of the intervention.

Weaknesses

- Recruiting participants <20 weeks gestation is necessary for the intervention to succeed and for outcomes assessment, but recruiting this early in pregnancy may be a challenge.
- Using social media as the primary recruitment strategy may result in some selection bias in the types of participants included in the study. Linking recruitment efforts to clinical care and having the community organizations help with recruitment might solve some of this risk for selection bias. The table in the Recruitment and Retention plan nicely shows some of the other strategies.

- There is some description of what will be in the prenatal technology platform (SMS messaging, wellness content) and where content will come from (BehavioralRX), but there is limited description of the development process – what has been done, what needs to be done, the process of creating/adapting content.

5. Environment:

Strengths

- The proposal appropriately points out that Nebraska is under-represented in NIH research, so this study include Nebraska Black women who have not been previously included in research
- The collaborators and supports needed foe the success of this study have been assembled and organized for this study.
- The research team has access to the needed supports and resources for conducting this study.

Weaknesses

- It is unclear the role some of the resources described in the Facilities and Resources section will have in supporting this project.

Study Timeline:

Strengths

- The timeline seems appropriate

Weaknesses

- Narrative supplementing the table would provide more insight into the activities shown in the study timeline table.

Protections for Human Subjects:

Acceptable Risks and/or Adequate Protections

- Thorough and well described plan

Data and Safety Monitoring Plan (Applicable for Clinical Trials Only):

Acceptable

- Excellent data and safety monitoring plan with defined adverse events and a DSMB.

Inclusion Plans:

- Sex/Gender: Distribution justified scientifically
- Race/Ethnicity: Distribution justified scientifically
- For NIH-Defined Phase III trials, Plans for valid design and analysis: Not applicable
- Inclusion/Exclusion Based on Age: Distribution justified scientifically
- Appropriate

Vertebrate Animals:

Not Applicable (No Vertebrate Animals)

- N/A

Biohazards:

Not Applicable (No Biohazards)

- N/A

Resubmission:

- N/A

Renewal:

- N/A

Revision:

- N/A

Resource Sharing Plans:

Acceptable

- Would like to see a formalized data sharing plan with community partners.

Budget and Period of Support:

Recommend as Requested

Recommended budget modifications or possible overlap identified:

- Detailed budget with costs appropriately accounted for. Based on costs, there is a lot of infrastructure that needs to be built including hiring and training 10 doulas and $125k to create the PARITY mobile technology platform in year 1. Allocating budget for engaging and communicating with the community is a strength.

CRITIQUE 4

[redacted]

Overall Impact: Led by an early-stage investigator, this proposal aims to conduct a randomized controlled trial assessing the effect of a multi-component intervention (PARITY program - community-based doula plus mobile digital health intervention) to improve maternal heath outcomes among Black women. Overall, this is a very strong proposal. Strengths include a strong conceptual framework; very

interesting strength-oriented approach that leverage the capabilities of individuals and the communities rather than focusing on deficits; novel combination of community health worker and mobile platform; prior work demonstrating feasibility of the mobile platform; and excellent partnerships with the Black community in Nebraska, including a Black doula community health worker program. The mixed-method trial design is also strong, looking not only at primary maternal outcomes, but also several explanatory factors along with assessment of barriers and facilitators. Weaknesses include: lack of details on the mobile platform design and features; lack of integration of the platform with clinical information systems; lack of sustainability/dissemination plan, considering that the mobile platform is a commercial tool. Nevertheless, these weaknesses are relatively modest compared to the strengths and do not substantially affect overall enthusiasm for the proposal. If successful, the intervention could be scaled to other Black communities in the US and produce a significant impact on maternal health disparities.

1. Significance:

Strengths

- Research on multi-faceted interventions that aim to improve Black women's maternal health is critical.
- Scientific premise. Strong evidence that wellness impacts maternal outcomes. Evidence from the investigators that simple wellness life style changes lead to improved outcomes. Evidence that community-based doula programs improve maternal outcomes.
- Plan for Enhancing Diverse Perspectives (PEDP): The proposal does an excellent job describing the factors that contribute to maternal morbidity and mortality. The focus on strengths rather than deficits is particularly commendable.
- Addresses integrated supportive care through multi-component approach that covers healthcare access, quality, and social conditions.

Weaknesses

- None noted by reviewer.

2. Investigator(s):

Strengths

- [redacted]
- [redacted]
- [redacted]
- [redacted]

Weaknesses

- None noted by reviewer.

3. Innovation:

Strengths

- Strengths-oriented instead of deficit-oriented approach leverages the strengths of the community and individuals to improve maternal health.
- Multi-component intervention combining doula-based CHW care with mobile technology in a synergistic way.
- Mixed methods approach including group concept mapping exercise.
- Intervention and study design guided by Theory of Maternal Adaptative Capacity, which focuses on the ability of individuals and communities to adapt, cope, and take advantage of opportunities when facing risks.
- PEDP: Strengths-oriented instead of deficit-oriented approach

Weaknesses

- None noted by reviewer.

4. Approach:

Strengths

- Combination of community-based CHW and technology-based mobile intervention
- Strong mixed-methods RCT design aims to look not only at heath outcomes (aim 1), but also explanatory mechanisms (empowered strengths, wellness behaviors), and barriers and facilitators (novel group concept mapping exercise).
- Preliminary work shows that mobile platform is feasible and was well-received by patients.
- Strengths-orientation vs. deficit approach. Intervention is designed to decrease risks and bolster adaptive capacity of individuals at the same time.
- Intervention combines virtual care with real life support, which may enhance scalability.
- Intervention customization based on participatory research through focus groups including diverse perspectives from community members, doulas, and patients.
- The PARITY platform tailors messages to each patient based on several factors. Patients can chat, call, or video call their doula. A detailed log of patient engagement with the app is collected.
- Solid approach to ensure intervention fidelity includes comprehensive training and multiple methods to monitor fidelity and intervention dose.
- Scientific premise: strong justification to focus on strengths-oriented approach as opposed to the historical deficit-oriented approach. Strong preliminary work demonstrating feasibility, acceptability, and potential cost-savings of the PARITY intervention.
- PEDP: Strong participatory research design with intervention adapted to the needs of the community. Concept mapping approach (Aim 4) also seeks input to help elicit explanatory factors as well as barriers and facilitators. Excellent conceptual framework guided by Theory of Maternal Adaptative Capacity. Connections between the mechanisms and maternal health outcome are very explicit and deliberate in the study design, with explicit hypotheses testing those mechanisms. Timelines of the PEDP are feasible.

Weaknesses

- The mobile platform lacks details about its design and functionality.
- Data such as medications and appointments need to be manually entered in the PARITY platform. There is no integration with the patient’s electronic heath record.
- Standalone nature of the PARITY platform may limit scalability and sustainability, which are not addressed in the proposal.
- Since the PARITY platform is based on a commercial product, it is unclear how this affect sustainability and disseminated beyond the scope of the trial. For example, who will keep ownership of the platform content that is created/adapted under this proposal?

5. Environment:

Strengths

- Malone Maternal Wellness (community-based Black doula program) is a key partner in the proposal. Their engagement may help ensure the long-term sustainability of the intervention beyond the trial.
- I Be Black Girls is a critical partner in the proposal, as they include many community partner organizations that can be critical for the proposed research. They host the Omaha Black Doula Association and have a strong focus on maternal health.
- The University of Nebraska offers several relevant resources for the project, including the the Niedfelt Nursing Research Center led by co-I Balas, the Center for Reducing Health Disparities, and the Great Plains IDeA Clinical and Translational Research.
- PEDP: Proposal includes key partnerships from the Black community. Nebraska has is underrepresented in NIH funding. Participation of Nebraska Black women in research is also underrepresented

Weaknesses

- None noted by reviewer.

Study Timeline:

Strengths

- Well thought out timeline is appropriate and feasible.

Weaknesses

- None noted by reviewer.

Protections for Human Subjects:

Acceptable Risks and/or Adequate Protections

Data and Safety Monitoring Plan (Applicable for Clinical Trials Only):

Acceptable

Inclusion Plans:

- Sex/Gender: Distribution justified scientifically
- Race/Ethnicity: Distribution justified scientifically

- For NIH-Defined Phase III trials, Plans for valid design and analysis: Not applicable
- Inclusion/Exclusion Based on Age: Distribution justified scientifically

Vertebrate Animals:
Not Applicable (No Vertebrate Animals)

Biohazards:
Not Applicable (No Biohazards)

Resubmission:
- NA

Renewal:
- NA

Revision:
- NA

Resource Sharing Plans:
Acceptable

Budget and Period of Support:
Recommend as Requested

THE FOLLOWING SECTIONS WERE PREPARED BY THE SCIENTIFIC REVIEW OFFICER TO SUMMARIZE THE OUTCOME OF DISCUSSIONS OF THE REVIEW COMMITTEE, OR REVIEWERS' WRITTEN CRITIQUES, ON THE FOLLOWING ISSUES:

PROTECTION OF HUMAN SUBJECTS: ACCEPTABLE

INCLUSION OF WOMEN PLAN: ACCEPTABLE

INCLUSION OF MINORITIES PLAN: ACCEPTABLE

INCLUSION ACROSS THE LIFESPAN: UNACCEPTABLE **MODIFIED FOR FEASIBILITY**

The inclusion across the lifespan is unacceptable. See comments from Reviewer 1.

COMMITTEE BUDGET RECOMMENDATIONS:
Budget modifications requested. See comments from Reviewer 1.

Footnotes for 1 R01 NR020707-01; PI Name: Mollard, Elizabeth K

NIH has modified its policy regarding the receipt of resubmissions (amended applications).See Guide Notice NOT-OD-18-197 at https://grants.nih.gov/grants/guide/notice-files/NOT-OD-18-197.html. The impact/priority score is calculated after discussion of an application by averaging the overall scores (1-9) given by all voting reviewers on the committee and multiplying by 10. The criterion scores are submitted prior to the meeting by the individual reviewers assigned to an application, and are not discussed specifically at the review meeting or calculated into the overall impact score. Some applications also receive a percentile ranking. For details on the review process, see http://grants.nih.gov/grants/peer_review_process.htm#scoring.
